# Recent advancements in the management of retinoblastoma and uveal melanoma

**DOI:** 10.12703/r/10-51

**Published:** 2021-05-28

**Authors:** Amy C Schefler, Ryan S Kim

**Affiliations:** 1 Retina Consultants of Texas, Houston, Texas, USA; 2 Blanton Eye Institute, Houston, Texas, USA; 3 McGovern Medical School, University of Texas Health Science Center at Houston, Houston, Texas, USA

**Keywords:** Ocular, tumor, uveal, melanoma, retinoblastoma, chemotherapy, radiotherapy, genetics

## Abstract

Retinoblastoma in children and uveal melanoma in adults can pose a serious threat to both vision and life. For many decades, enucleation was often the only option to treat these intraocular malignancies. For retinoblastoma, intra-arterial chemotherapy is often utilized as the primary treatment at advanced academic centers and has dramatically improved local tumor control and eye salvage rates. For uveal melanoma, both plaque brachytherapy and proton beam irradiation have served as widely utilized therapies with a local failure rate of approximately 1–10%, depending on the series. Major recent advancements have allowed for a better understanding of the genomics of uveal melanoma and the impact of certain mutations on metastatic susceptibility. Gene expression profile stratifies uveal melanomas into two classes: low-risk (class 1) and high-risk (class 2). A loss-of-function mutation of *BAP1* is associated with a class 2 gene expression profile and therefore confers worse prognosis due to elevated risk of metastasis. On the other hand, gain-of-function mutations of *EIF1AX* and *SF3B1* correspond to a gene expression profile of class 1A and class 1B and confer a better prognosis. Preferentially expressed antigen in melanoma (PRAME) is an antigen that increases metastatic susceptibility when expressed in uveal melanoma cells. In addition to plaque brachytherapy and proton beam irradiation, both of which have demonstrated superb clinical outcomes, scientists are actively investigating newer therapeutic modalities as either primary therapy or adjuvant treatment, including a novel nanoparticle therapy and immunotherapy.

## Introduction

Eye cancer is a rare but potentially life-threatening disease. Although there are various kinds of intraocular malignancies, retinoblastoma and uveal melanoma are the most common primary intraocular malignancies. Retinoblastoma primarily occurs in children, with 250–350 cases per year in the United States almost exclusively in patients under 5 years of age^1^. Uveal melanoma is the most frequently observed primary intraocular cancer in adults, with 5.1 cases per million mostly seen in Caucasians^2^. In this review, we will provide updates on treatment guidelines, newly emerging therapeutic modalities, areas of research focus, and future directions for the management of retinoblastoma and uveal melanoma.

## Retinoblastoma

Retinoblastoma is rare in the general population but is the most common primary pediatric intraocular malignancy. Retinoblastoma occurs when both alleles of the *RB1* gene, which is a tumor suppressor gene located at chromosome 13q14, are mutated. Approximately one-third to 40% of retinoblastoma is attributable to a mosaic or germline mutation of the *RB1* gene^3^, while sporadic cases account for more than 50% (Table 1). Almost 90% of the germline cases are due to a new mutation with no familial history of retinoblastoma^3^. Retinoblastoma can present as either unilateral or bilateral disease, which accounts for approximately two-thirds and one-third of all cases, respectively. More than 80% of unilateral retinoblastoma arises from a sporadic mutation and 15% due to a germline mutation^4^. Less than 2% of unilateral, non-familial retinoblastoma patients may present with fully functional *RB1* but instead with a *MYCN* oncogene mutation. Patients with *MYCN* mutations present early at a median age of 4 months with an aggressive course^5^.

**Table 1.** Comparison of characteristics between sporadic and hereditary retinoblastoma.

	Sporadic	Germline	Germline-mosaic
Number of mutated cells	One	All	Variable
Laterality	Always unilateral	85% bilateral, 15% unilateral	Either unilateral or bilateral
Average age at presentation	18~24 months	12~18 months	Variable
Chance of inheritance to offspring	0%	45%	Variable

In contrast to unilateral cases, bilateral retinoblastoma is exclusively due to a germline mutation, which may be present already in the germ cell or can occur *de novo* during early embryogenesis. In the former, every cell in the body will carry the mutation, whereas the latter will cause mosaicism in which a proportion of cells are mutated. In either case, there is increased risk of malignancy in other parts of the body during the lifetime of the patient, including soft tissue sarcomas, osteosarcoma, and cancers of epithelial origin^6,7^. It is believed that germline retinoblastoma can also lead to an approximately 5% risk of an intracranial malignancy, most commonly pineoblastoma. Many centers perform routine monitoring with imaging even after successful intraocular treatment at least up until 5 years of age to evaluate for this second malignancy^8^. Recent controversial evidence suggests that secondary central nervous system (CNS) tumors occur in only about 5% of patients with germline mutations, especially if the patient did not receive any form of radiotherapy. Some centers have transitioned away from routine CNS surveillance after retinoblastoma treatment because of a low yield of detecting CNS tumors post-treatment^9^. However, it should be noted that pineoblastoma and trilateral retinoblastoma, which are of particular concern for germline retinoblastoma patients, were excluded from analysis. Therefore, without a large prospective study, it is premature to recommend against no routine CNS surveillance after standard retinoblastoma treatment.

With major advancements in retinoblastoma treatment in the past two decades including intra-arterial chemotherapy (IAC) and intravitreal chemotherapy (IvitC), advanced academic centers across the globe have achieved over 95% long-term disease-free survival and 85% long-term globe salvage, which will be discussed further below^10^.

### Retinoblastoma classification and current mainstay treatment options

Both Reese-Ellsworth and the International Classification of Retinoblastoma (ICRB) systems can be used to classify retinoblastoma (Table 2). However, the Reese-Ellsworth classification was introduced decades ago when external beam radiation therapy (EBRT) was primarily used, and the ICRB system (in which tumors are grouped into A through E) has largely replaced its older counterpart in current ocular oncology practice^11^. Ideally, the goal of retinoblastoma treatment is to cure the patient and secondarily preserve the globe and vision when possible. For ICRB group A, B, and some C tumors, focal therapy with laser ablation, cryotherapy, or transpupillary thermotherapy (TTT) may be sufficient to achieve full local tumor control^12^. On the other hand, group C tumors involving the macula or group D or E need to be more aggressively treated. Intravenous chemotherapy (IVC) using vincristine, carboplatin, and etoposide has been successfully used for decades to reduce the size of the tumor followed by focal therapies, with its efficacy demonstrated in multiple publications^13–15^. IVC can achieve over 90% of tumor control for group A–C tumors and is also frequently used to treat group D retinoblastoma, albeit with lower ocular salvage rates^15,16^. Group D or E tumors with high-risk features, including concern for extraocular extension of the tumor to the surrounding structures during examination under anesthesia, optic nerve, or choroidal invasion, are generally enucleated to reduce the risk of metastasis^17–19^. Shields *et al*. recently published a 20-year outcome of IVC therapy for retinoblastoma, in which groups A, B, and C achieved over 90% tumor control rate, while groups D and E achieved 71% and 32% tumor control, respectively^18^. Notably, less than 2% of the patients experienced metastasis and 1% died at 20 years of follow-up, demonstrating excellent long-term disease control. In addition to advanced unilateral disease, IVC can also be used to manage bilateral retinoblastoma with a germline *RB1* mutation.

**Table 2.** Summary of International Classification of Retinoblastoma (ICRB) classification.

International Classification of Retinoblastoma
Group A – tumors <3 mm and away from fovea and optic disc
Group B – tumors >3 mm, located at macula/peripapillary region, or with subretinal fluid
Group C – tumors with focal vitreous or subretinal seeds within 3 mm of tumor
Group D – tumors with diffuse vitreous or subretinal seeds >3 mm away from tumor
Group E – tumors covering >50% of globe with or without neovascular glaucoma, hemorrhage, or extension of tumor to optic nerve/anterior chamber

**Figure 1.**
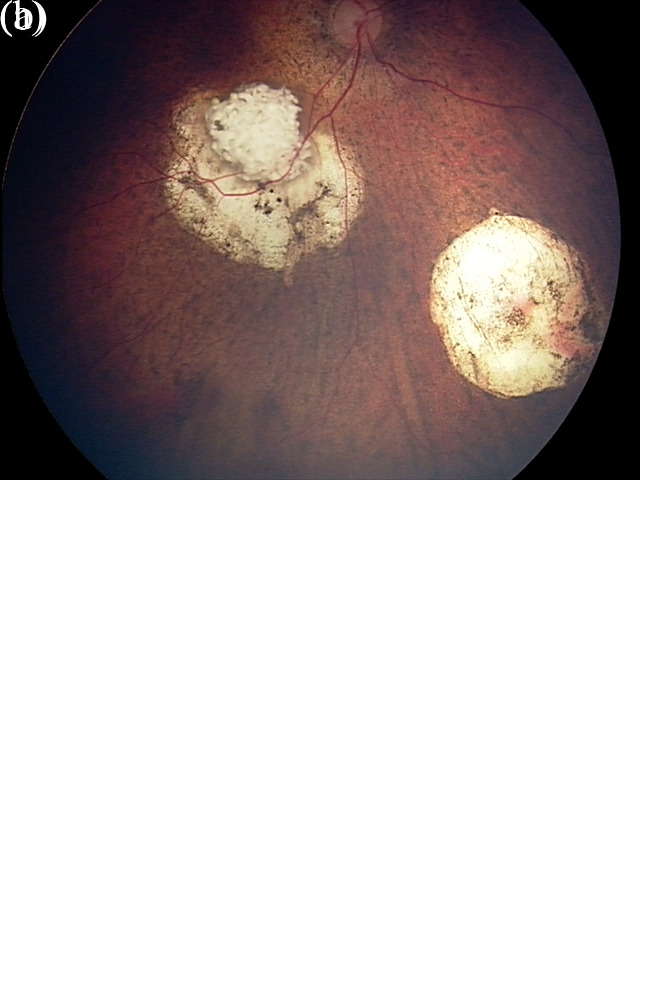
Retinoblastoma of a 5-month-old patient before and after intra-arterial chemotherapy (IAC). (**a**) Fundus photograph of the right eye before IAC demonstrating macular and inferonasal lesions. (**b**) Fundus photograph of the same eye 13 months after the initial IAC treatment. The patient underwent three IAC cycles and adjuvant therapy, including five sessions of laser ablation and two sessions of cryotherapy. This figure was reused from Schefler A and Kim R^49^ under the terms of CC BY 4.0 (https://creativecommons.org/licenses/by/4.0/).

Since the early 2000s, IAC has emerged as a promising alternative for IVC and enucleation (Figure 1). Not only is IAC a globe-salvaging treatment, but it has also demonstrated superior local tumor control with significantly fewer systemic toxicities, including immunosuppression, ototoxicity, and nephrotoxicity, than IVC^16,20–28^. In particular, for group D tumors, which had been conventionally treated with IVC or enucleation, numerous studies using IAC as primary therapy have reported 85–90% globe salvage rates^16,21,29,30^. Therefore, large academic centers with experienced ocular oncologists and neuro-interventionalists now prefer to utilize IAC as first-line treatment for most cases of retinoblastoma. Moreover, IAC has demonstrated efficacy as secondary treatment. Over 80% and 50% ocular event-free survival has been observed for patients who underwent IAC as secondary therapy after failing IAC^31^ or IVC and EBRT^32^, respectively.

For very young patients, usually under 6 months of age, either bridge therapy or IAC is currently used. In bridge therapy, IVC is performed at the time of diagnosis for initial disease control until the patient reaches a weight at which IAC can be safely administered (typically 6 to 10 kg)^33^. This convention is to minimize procedure-related complications in small infants, such as catheterization site hematoma and limb ischemia. However, more studies are demonstrating that IAC can be safely administered in infants weighing less than 10 kg, with the youngest reported patient only 35 days of age when receiving the first cycle of IAC^34–38^. Sweid *et al*. published a comparative study in which patients weighing less than 10 kg experienced fewer total IAC cycles as well as less exposure to radiation during fluoroscopy, which can help minimize procedure-related adverse effects in the long term^34^. As a result, there is increasing understanding that younger patients undergoing IAC as primary therapy may attain better outcomes in a shorter treatment duration^29,34,39^.

For ICRB groups C, D, or E retinoblastoma with recurrent or persistent vitreous seeds after primary therapy (either IVC or IAC), IvitC is often utilized^40,41^. The technique is performed with measures to minimize needle tract tumor seeding after injection and is now widely used for various types of vitreous seeds. Previously, vitreous seeds were considered higher risk and often warranted enucleation because both IAC and IVC had limited bioavailability within the vitreous and therefore had limited efficacy. In contrast, IvitC allows for direct injection of the chemotherapeutic agent into the intraocular space and has achieved excellent seed control in multiple studies^42–44^. It has therefore reduced the rate of enucleation due to persistent vitreous seeds in many cases. Rates of ocular salvage in many recent publications reflect the combination use of IvitC and IAC and thus may not reflect results that would be obtained with IAC alone. Melphalan is the most commonly used agent and usually achieves excellent vitreous seed control alone. However, if there is concern for resistance to melphalan, topotecan may be added simultaneously or separately to augment intravitreal therapy^17^. In some instances, seeds from the primary retinoblastoma lesion may disseminate to the anterior chamber. Previously, enucleation was usually warranted when tumor seeding in the aqueous humor was observed. There are reports of successfully controlling aqueous seeds with intracameral injection of melphalan, but larger studies are needed to further validate the efficacy of intracameral chemotherapy^45–47^.

### Factors that affect primary treatment choices and treatment outcomes

There are several variants of the ICRB that are currently used, including the Children’s Oncology Group Philadelphia^48^ and Children’s Hospital of Los Angeles versions^11^. The major implication of using different classification systems is that tumors may fall under different classes depending on which system is used, and corresponding treatment choices therefore may not be homogeneous. This is particularly true for group D tumors, whose definitions vary the most. To add another dimension of complexity, treatment choices are also influenced by the financial and medical resources that are available to the ocular oncologist and the patient. IAC has been demonstrated to be an excellent treatment modality with superior tumor control and globe preservation. Numerous studies suggest that IAC can outperform IVC for advanced tumors belonging in groups C and D and help avoid enucleation^21,23^. However, for successful IAC, it often requires a multidisciplinary approach including expert ocular oncologists and neuro-interventionalists, who are not ubiquitously available, especially in less-developed countries, from which more than 80% of the total retinoblastoma patients originate^50^. Such disparities lead to limited availability of IAC to only a fraction of the patients. More than half of the physicians in a global survey of retinoblastoma experts by Scelfo *et al*. reported using IVC as primary treatment for group D tumors, while 27% perform primary enucleation, followed by only 16% who offer IAC as first-line therapy^51^.

In addition to the issue of availability, enucleation and IVC may sometimes make more financial and logistical sense for patients. Enucleation is significantly less costly than IAC and requires less frequent follow-up. Fabian *et al*. reported that fewer exams under anesthesia (EUA) are required for group D patients whose eyes were enucleated compared to those who underwent IAC^52^. For patients who have difficulty receiving regular check-ups for various reasons, enucleation may serve as a better alternative.

Studies have demonstrated that there exists a significant difference in treatment outcomes for retinoblastoma between advanced and less-developed nations. In a recent cross-sectional study of almost 280 retinoblastoma centers around the globe, retinoblastoma was diagnosed significantly later (30 months vs. 14 months) in less-developed countries, and patients often had advanced disease with extraocular extension of the tumor (49%) as well as metastasis (19%), whereas these were much less frequently observed in advanced nations (1.5% extraocular extension and 0.3% metastasis) at the time of diagnosis^10,53^. Unfortunately, as a result, treatment outcomes are usually significantly worse owing to late presentation in developing countries, and mortality rates are as high as 60–70% in some nations^54–58^. Considering how major advancements in retinoblastoma research have greatly shifted the paradigm of the treatment of retinoblastoma and have made it a largely treatable and curable cancer in advanced nations, there have been efforts to mitigate the mortality and enucleation rates in less-developed countries. Sharing of resources and adequately educating patients and their families about retinoblastoma and its treatment options are some of the ongoing efforts, but there is still certainly a large gap to close^57^.

### Genomic analysis of retinoblastoma

As germline retinoblastoma accounts for approximately 30–40% of all retinoblastoma cases, it is important to determine for each patient whether he/she has a mosaic or germline mutation and therefore possesses a risk for secondary malignancies in other parts of the body as well as the possibility of retinoblastoma in other family members. In cases of bilateral disease, a germline mutation can be assumed, but it is inadvisable in cases of unilateral disease to assume a somatic mutation. Studies have shown that approximately 15% of unilateral cases are due to germline mutations, and they can subsequently develop tumors in the unaffected eye^59^. Therefore, early genotyping can provide crucial insights into what type of retinoblastoma the patient has, enabling optimal treatment plans and genetic counseling^60^. Recently, there has been a potentially exciting development of cell-free DNA analysis of aqueous humor samples that can detect mutations in tumor cells with a high accuracy rate. A small amount of aqueous humor aspirate, as little as 0.1 ng/μl, can help detect *RB1* mutations and determine tumor zygosity based on mutated *RB1* allele frequency^61–63^. Moreover, Gerrish *et al*. reported that more than 90% of the cell-free DNA sample in the acquired aqueous humor originates from the tumor itself, and the amount of DNA content correlates specifically with the tumor burden^61^. Once this molecular testing method is validated in larger multicenter studies, cell-free DNA analysis of retinoblastoma may allow physicians to precisely identify germline *RB1* or *MYCN* mutations and prepare personalized therapy regimens and family genetic counseling.

### New therapeutic targets and future directions

For the past two decades, active research has been undertaken to identify new molecular targets specific for retinoblastoma. Some recent publications have identified several genes and pathways that may hold the key for retinoblastoma tumorigenesis and potential therapy options. There is growing evidence that retinoblastoma cells and retinal cone precursor cells may share common pathways for proliferation. Previously, it has been observed that retinoblastoma cells require suppression of p27, which is regulated by increased SKP2 expression^64^, and also that cone precursor cells have downregulated p27^65^. Moreover, it has been observed that, in order to escape apoptosis and further proliferate, retinoblastoma cell proliferation depends on the expression of proteins including RXRγ, MYCN, MDM2, and TRβ2, which are also greatly produced by retinal cone progenitor cells^66^. Xu *et al*. published that when retinal cone progenitor cells undergo a *RB1* gene knock-out, they exhibit significantly increased proliferation and dependence on MYCN, MDM2, and SKP2 expression, which is very similar to how retinoblastoma cells proliferate^67^. Together, it is postulated that retinoblastoma may possibly originate from cone precursors and that targeting some of the key cone precursor cell signaling pathways may allow us to gain insights into specific gene-targeted therapy for retinoblastoma. Other molecular targets such as the spleen tyrosine kinase^68^ and orthodenticle homeobox 2 (OTX2)^69^ are also being investigated.

With increasing emphasis by researchers on the molecular understanding of retinoblastoma, significant advancements are anticipated in the coming decade.

## Uveal melanoma

Uveal melanoma, although rare, is the most common primary intraocular cancer in adults, with an incidence rate of 5–7 cases per million or approximately 2,000 new cases per year in the United States^2^. Uveal melanoma most frequently presents unilaterally in the sixth or seventh decade of life and has a heavy predominance in the Caucasian population^2^. Very rarely, patients with germline *BAP1* mutations can develop bilateral primary uveal melanoma^70^. Current mainstays of treatment are plaque brachytherapy (most commonly iodine-125 in the United States and ruthenium-106 in Europe and other nations) or proton beam irradiation (PBI). Advanced cases may require enucleation, while some localized iris tumors, which account for only 3–5% of all cases, may be surgically excised^71^. While the overall 5-year survival rate has remained close to 80%^2,72^, up to 60% of uveal melanoma patients develop metastatic disease despite timely treatment and consistent follow-up^73,74^. This is thought to be due to early hematogenous spreading of the tumor cells that are often difficult to detect with current diagnostic capacities^75^. Liver, lung, and bone are the most common organs to which uveal melanoma metastasizes, and once metastatic disease is identified, there is not yet an effective treatment that can meaningfully lengthen survival in all patients^73,74,76–79^.

### Genomic analyses of uveal melanoma

Numerous studies have helped us better understand how uveal melanoma develops and how it differs from a choroidal nevus, which is a benign intraocular lesion that is commonly seen in up to 10% of the population, especially among Caucasians^80^. Most nevi are found in the posterior half of the eye and remain stable in size or grow very slowly over many years^81^. On the other hand, choroidal melanoma appears to undergo several critical genomic and molecular changes that are quite different from the mutations seen in cutaneous melanoma, such as the *BRAF* mutation^82^. The pathogenesis of uveal melanoma usually involves an initiating mutation in the Gα11/Q pathway, which activates multiple downstream pathways including the MAPK and YAP^83^ pathways. Choroidal nevi have *GNAQ* or *GNA11* mutations too, but uveal melanoma further undergoes one of three key driver mutations as part of malignant transformation: *BAP1, SF3B1*, or *EIF1AX*. *BAP1* is a tumor suppressor gene located on chromosome 3 that plays a role in protein de-ubiquitination, cell cycle regulation, and DNA repair^84,85^. Specifically, BAP1 interacts with genes that are critical for maintaining the differentiated state of the cell, and a *BAP1* mutation can thus make the cell become more stem cell-like and ultimately increase the risk of metastasis in uveal melanoma^86^. Either monosomy 3 or a somatic knock-out of the second *BAP1* allele leads to *BAP1* inactivation in approximately 35–45% of all uveal melanomas and confers the worst prognosis of these three key driver genes^87,88^. *SF3B1* is a splicing gene whose mutation accounts for approximately 20–25% of uveal melanoma cases^87,89^. When a mutation occurs in the *SF3B1* gene, which normally codes for the U2 snRNP complex of the spliceosome, frameshift insertions may occur and lead to incorrect splicing of the pre-mRNA and subsequent mRNA degradation^89,90^. It is, however, not yet fully understood how this spliceosome aberration is linked to the malignant transformation of uveal melanoma. Lastly, *EIF1AX* is a gene that encodes the eIF1A initiation factor, which is a component of the 43S preinitiation complex that is involved in the early steps of translation initiation. Mutations in *EIF1AX,* seen in approximately 20–25% of tumors, lead to amino acid substitutions or deletions in the N-terminal tail and interfere with proper translation^88,91^. In most cases, mutations of these three key genes occur in a mutually exclusive fashion, and the specific prognostication differs based on which mutation the tumor harbors.

Multiple studies have suggested that *BAP1, EIF1AX,* and *SF3B1* mutations are linked to other prognostic indicators. Gene expression profiling (GEP), which quantifies the mRNA expression of 12 key genes (and three control genes), classifies uveal melanoma as either class 2 with high metastatic risk or class 1 with lower metastatic risk^92,93^. Class 1 tumors are further subdivided into class 1A, which carries a 2% risk of metastases at 5 years, and class 1B, which carries a 21% risk of metastases at 5 years^94^. Although class 1B tumors do not metastasize as aggressively as class 2 tumors, which have a 72% incidence of metastatic disease at 5 years, 1B tumors are thought to carry a gradually increasing metastatic risk over time^94^. Therefore, close monitoring of class 1B and class 2 tumors with abdominal imaging is recommended^94^. Current data suggest that *BAP1* is predominantly seen in tumors with a GEP class 2 signature, loss of chromosome 3, and gain of chromosome 8q and leads to significantly worse prognosis^95^. *SF3B1* is more closely associated with class 1B tumors and leads to late-onset metastasis, conferring intermediate risk. *EIF1AX* mutations are generally seen in patients with a class 1A signature and are associated with a lower metastatic risk^94,96^. Therefore, once uveal melanoma is diagnosed, utilization of prognostic molecular testing can help guide the patient to receive personalized disease monitoring.

Although our understanding of primary uveal melanoma has greatly improved over the past two decades owing to major advancements in genomic and molecular research, there is still much to learn about metastatic uveal melanoma because no new therapies have decisively reduced the mortality rate. Nonetheless, recently, there have been several new and potentially controversial reports on the genomics of the disease. Shain *et al*. reported that some metastatic lesions could potentially develop even before key chromosomal or genomic changes occur in the primary tumor, including the 8q gain or loss of both *BAP1* alleles^97^. In the metastatic lesions, new oncogenic changes, such as *CDKN2A* loss, *MBD4* deficiency, and *EZH2, PTK2B,* and *PBRM1* gene mutations, were observed^97–99^, indicating that primary and metastatic uveal melanoma may have quite different genetic compositions. This data has not been validated on a larger scale in additional laboratories.

### Plaque brachytherapy and proton beam irradiation

**Figure 2.**
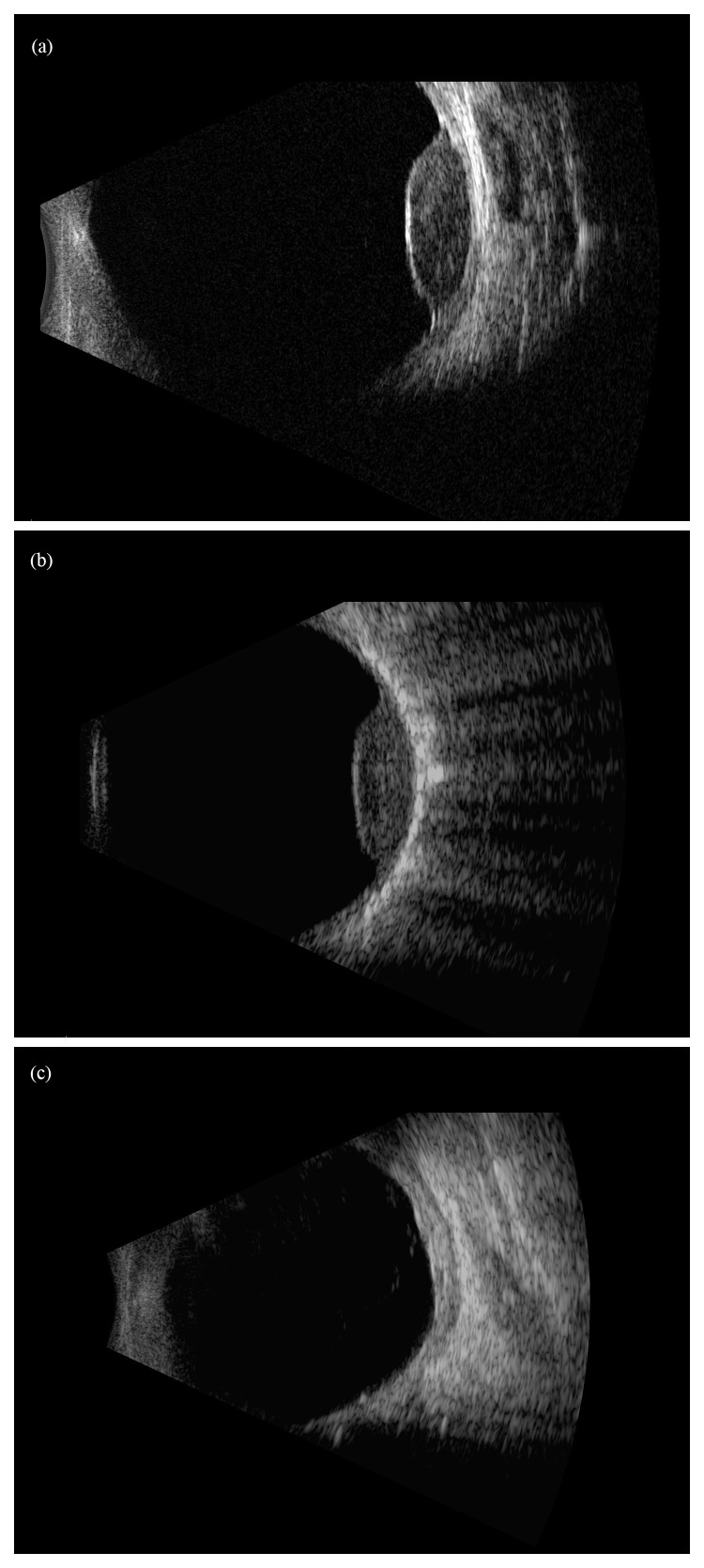
Uveal melanoma of a 66-year-old patient before and after plaque brachytherapy. (**a**) B-scan ultrasound image of the right eye before the plaque implantation. (**b**) B-scan ultrasound image of the same eye intra-operatively, demonstrating full coverage of the tumor with the plaque. (**c**) B-scan ultrasound image of the same eye 3 years after the plaque therapy, demonstrating regression of the tumor. This figure was reused from Schefler A and Kim R^48^ under the terms of CC BY 4.0 (https://creativecommons.org/licenses/by/4.0/).

The Collaborative Ocular Melanoma Study (COMS) published a series of landmark studies on uveal melanoma since 1990 that laid the cornerstone for diagnosis and management of uveal melanoma. The pivotal COMS medium tumor trial compared the efficacy of iodine-125 plaque brachytherapy with that of enucleation and concluded that there was no statistically significant difference in survival between patients who underwent plaque brachytherapy and patients who underwent enucleation^100^. Since then, most centers have adopted plaque brachytherapy as the standard treatment for uveal melanoma, and iodine-125 brachytherapy is the most commonly used treatment modality for uveal melanoma in the United States with excellent clinical outcomes (Figure 2). Outside the United States, ruthenium-106 and palladium-103 are also frequently used^101^. Iodine-125 and palladium-103 are both gamma ray emitters with a lower energy profile, while ruthenium-106 emits beta rays and has a steeper dose fall-off^102^. With the steeper dose fall-off, ruthenium-106 may cause less radiation-induced damage to the surrounding normal structures that are important for the preservation of vision, including the macula and optic disc, whereas iodine-125 can be effective for thicker tumors (>5 mm in height) for which ruthenium-106 may not be adequate to achieve local control^103^.

PBI has also been used as primary therapy for uveal melanoma for several decades. Tantalum beads (MRI-safe metal) are surgically attached to the sclera, and the beads can remain in the orbit permanently, even after PBI. A series of PBI sessions occur over several days with a total cumulative radiation dosage of 50 to 70 Gray (Gy)^104^. In addition to small and medium-sized tumors, PBI can be particularly effective for patients with large tumors that sometimes may not be ideal candidates for plaque brachytherapy owing to size limitations, albeit typically with severe long-term radiation complications. Papakostas and colleagues published a single center cohort study of patients with large tumors (mean largest basal diameter [LBD] of 18.1 mm and thickness of 8.2 mm) who were treated with PBI; 10-year eye retention rate was 70.4%, and tumor control rate was 87.5%^105^.

At advanced centers, PBI and plaque brachytherapy have comparable tumor control rates, with multiple recent publications reporting local recurrence rates of 5% or less for both treatment modalities^106–112^. Some reports even demonstrated no local treatment failure for up to 2 years post-brachytherapy^110^, which is a significant improvement from the 10.3% local recurrence rate at 5 years after brachytherapy that was originally reported in the COMS publications^113^. Achieving long-term local tumor control is critical because the probability of the primary lesion metastasizing significantly increases once tumor recurrences are observed^111,114^. One study by Bellerive *et al*. reported a local recurrence rate of 5.6% with a median onset of 18 months after initial brachytherapy^108^. In this study, one-third of the patients with local recurrences underwent repeat brachytherapy with no further evidence of recurrence afterwards during a median follow-up of 45 months. In another study by Riechardt *et al*., salvage PBI was performed for 48 patients who developed local recurrences after the first therapy, with 92.1% local control at 10 years of follow-up^115^. However, even though local control was achieved in most cases, both studies had a 30–50% rate of metastatic disease over 10 years, demonstrating again that the risk of metastatic mortality is significantly elevated when local treatment failure occurs.

Several key factors have contributed to improved treatment outcomes for both PBI and plaque brachytherapy. Detailed imaging studies including fundus photographs, computed tomography (CT), and ultrasonography allow for accurate determination of the tumor anatomy, tumor location, as well as pre-treatment 3D planning^116^. Based on this information, radiation dosage simulation is performed, and potential radiation exposure to the critical structures including the optic nerve and macula is also calculated^104,110^. In addition to the 3D simulation, intraoperative ultrasound confirmation of the plaque placement, which has been in use for the past two decades^117–119^, serves a critical role to ensure that all tumor margins are fully covered by the plaque that is newly inserted. Aziz *et al*. published a retrospective study in which the patients who underwent intraoperative ultrasonographic confirmation of the plaque position experienced a 1.5% treatment failure in contrast to the 9.3% in those who did not^120^. For plaque brachytherapy, thinner and customizable designs also help conform to each tumor’s specific geometry and offer more targeted therapy while minimizing radiation damage to critical ocular structures^121–123^.

### Radiation retinopathy

Patients undergoing PBI or plaque brachytherapy are at risk of developing radiation-related ocular complications based on which ocular structures the radiation beam traverses. Anterior segment complications include cataract, rubeosis iridis, and neovascular glaucoma^124^. Posteriorly, optic neuropathy and radiation retinopathy are common. Radiation damages capillary endothelial cells and pericytes, which then causes areas of retinal capillary nonperfusion^125^. Severe retinal ischemia can then subsequently lead to neovascularization and retinal hemorrhage as well as microaneurysms that can affect visual acuity. Tumors that are within 4 mm of the fovea or larger than 10 mm in LBD are at significant risk for radiation maculopathy^126^. As high as 50% of patients undergoing radiotherapy for uveal melanoma may develop OCT- or OCT-A evident macular edema, which is an early sign of radiation retinopathy that typically manifests between 1 and 2 years post-radiotherapy^127–129^.

Treatment options for radiation retinopathy include laser photocoagulation, intravitreal anti-VEGF, and steroids. Most studies investigating the use of intraocular steroids have been limited by small sample sizes and lack of randomization^130–133^. On the other hand, multiple studies have investigated the use of intravitreal anti-VEGF and demonstrated efficacy for preserving best-corrected visual acuity (BCVA). In addition to numerous retrospective studies reporting on improved macular edema and central macular thickness (CMT) after the use of anti-VEGF therapy, several key prospective studies have shown statistically significant improvement in BCVA after regularly injecting intravitreal anti-VEGF. Murray *et al*. reported that 39 patients who received aflibercept injections either at a fixed 6-week interval or via a treat-and-extend protocol after developing post-radiotherapy radiation maculopathy showed improvements in BCVA at 1 year follow-up, with 42.5% having better than 20/50 vision, as well as enhanced CMT^134^. Fallico, Russo, and colleagues, despite a small sample size of nine eyes, also reported similar improvements in BCVA and CMT after administering a mean of 4.4 aflibercept injections over 2 years^135^. Schefler *et al*. published results of a prospective, multicenter ranibizumab therapy trial for clinically evident macular edema after iodine-125 plaque brachytherapy^136^. The study compared a monthly ranibizumab injection group to two other groups: one who received targeted pan-retinal laser photocoagulation (TRP) with monthly ranibizumab and another group that received as-needed ranibizumab injections (PRN) with TRP. At 1 year, the monthly injection group demonstrated significantly better BCVA against the monthly injection plus laser group as well as the PRN group (+4.0 Early Treatment Diabetic Maculopathy Study [ETDRS] letters in BCVA vs. –1.9 letter vs. +0.9 letters, respectively, *P* <0.001). A total of 82% of the patients maintained 20/200 or better BCVA at 1 year, while 20% gained 10 or more ETDRS letters, all of which indicated significantly better visual outcomes compared to historical controls^137^. Similarly, Seibel *et al*. reported that patients who received four to six ranibizumab injections in the first 6 months after developing radiation maculopathy had significantly better BCVA than a control group that received pan-retinal laser photocoagulation to the ischemic retina^138^. Kim *et al*.^139^ published a prospective study in which 40 patients received prophylactic ranibizumab every 2 months for 2 years starting 2 weeks prior to PBI therapy for uveal melanoma. In this study, 88% of patients maintained 20/40 or better visual acuity at 2 years of follow-up, despite the fact that all of the study patients had tumors within two disc diameters of the macula or the optic nerve and therefore had high risk for developing radiation-induced maculopathy or papillopathy.

The exact timing of anti-VEGF therapy initiation for radiation retinopathy is not yet known, as both prophylactic injections^139,140^ and injections once clinically evident macular edema manifests^128,136^ have demonstrated clinical efficacy. Nonetheless, the current general consensus is that, in order to effectively maintain or improve visual acuity and reduce macular edema, long-term anti-VEGF injections need to be administered on a regular basis.

### Clinical and molecular features with prognostic significance

Molecular analysis methods including the gene expression profile (GEP) and multiplex ligation-dependent probe amplification (MLPA) can give valuable prognostic information by identifying key genomic mutations and corresponding metastatic potential. Various clinical risk factors, when combined with the genomic classifications, may strengthen the prognostic predictions for metastatic disease in uveal melanoma. Tumor size has long been understood to be a key factor for predicting outcomes. The LBD can serve as an independent marker for predicting metastasis^141,142^. Binkley *et al*. reported that various tumor size classification systems, including the American Joint Committee on Cancer (AJCC), COMS guidelines, tumor LBD, and tumor thickness, can reinforce the prognosis when combined with the GEP result compared to the GEP result alone^143^. Berry *et al*. stratified 360 melanoma patients into AJCC stages 1, 2, and 3 as well as GEP class and discovered that there was a significant correlation between larger tumor dimensions (both LBD and thickness) and class 2 status (*P* <0.05)^144^. Moreover, larger AJCC tumor groups had a significantly higher odds ratio of having a worse prognosis based on the corresponding GEP class. Walter *et al*.^145^ reported that patients with class 2 uveal melanoma had a significantly lower 5-year metastasis-free survival when the tumor was larger than 12 mm in LBD, which was the same LBD threshold for a worse prognosis in a study by Demirci *et al*.^142^.

Preferentially expressed antigen in melanoma (PRAME) is a cancer-testis antigen that is not normally produced by healthy human tissues but is expressed in different types of cancer^146^. PRAME helps cancer cells to survive by suppressing retinoic acid-driven apoptosis and inhibition of cell proliferation^147^. Field *et al*. reported that increased PRAME mRNA expression is observed in uveal melanoma and that GEP class 2 tumors are more likely than the class 1 signature to be PRAME+^148^. Furthermore, PRAME+ class 2 tumors progress faster to metastases, and PRAME+ class 1 tumors are more likely to metastasize than PRAME– tumors^149^. Newer publications have investigated possible relationships between the PRAME status and other prognostic factors, and more studies on this subject are in progress. Cai *et al*. reported that the combined PRAME status and GEP information can offer a higher prognostic accuracy than optimized TNM staging alone^150^. Schefler *et al*. conducted a retrospective study in which PRAME status was analyzed in accordance with other clinical and molecular factors^151^. In 148 uveal melanoma patients who underwent PRAME testing, 37% of the patients were positive for PRAME and subsequently had a statistically significant association with a worse GEP classification. More than half of GEP class 2 tumors were PRAME+, while approximately 30% of the class 1 tumors were PRAME+. Given that certain class 1A tumors still progress to metastatic disease and that PRAME+ appears to be associated with increased metastatic disease rates, a positive PRAME expression status in a GEP class 1 tumor may explain the rare cases in this group that do metastasize. Future studies will help identify additional associations between PRAME and other more established risk factors for metastasis.

There has been a debate on whether tumor response to radiotherapy can be predicted based on the GEP classification. While several clinicians have reported no significant difference in the rate of regression between class 1 and class 2 tumors at 3 and 6 months after iodine-125 brachytherapy^152,153^, Rao *et al*.^154^ and Mruthyunjaya *et al*.^155^ observed significantly greater reductions in tumor thickness in class 1 tumors at 3 and 6 months post-therapy and 3 months post-therapy, respectively. More data are needed on this subject to elucidate a clearer relationship.

Recently, Liu *et al*. published an interesting pilot study introducing deep machine learning to correlating histopathologic significance to the GEP classification^156^. In this study, machine learning assessed 10 hematoxylin-eosin pathology slides from GEP class 1 tumors and 10 from class 2 tumors based on classic tumor features: spindle-type cells for lower-grade tumors and epithelioid-type cells with high degree of atypia for higher-grade tumors. Interestingly, 15 of the 20 slides were correctly analyzed and classified as either GEP class 1 or 2. Although the data from this study are limited and preliminary, this result suggests that deep machine learning can potentially be applied in various ways to assist clinicians with predicting metastatic risk in uveal melanoma.

### Other therapies

Because our understanding of the molecular genetics of uveal melanoma has seen rapid advancements in the past decade, clinical trials utilizing our understanding of these pathways have begun. Durante and colleagues recently reported that tumor-infiltrating T cells in uveal melanoma express not just PD1 and CTLA4 checkpoint markers but also LAG3, which plays a critical role in T cell exhaustion and subsequent immune escape^157^. In their study, class 2 tumors predominantly expressed LAG3, not PD1 or CTLA4, which may account for why checkpoint inhibitor therapy targeting PD1 and CTLA4 fails to work in many cases. Faião-Flores *et al*. investigated various cell signaling pathways in uveal melanoma and observed YAP and AKT upregulations when the MEK pathway was inhibited, explaining the increased resistance by uveal melanoma cells upon MEK inhibition^158^. Interestingly, when panobinostat, a histone deacetylase (HDAC) inhibitor, was added to the MEK inhibitors, the drug appeared to limit the MEK resistance by downregulating the YAP and AKT signaling. If further studies and clinical trials show promising outcomes, immunotherapy that targets the LAG3 checkpoint or the HDAC/MEK pathway may provide new insights into uveal melanoma treatment. Such trials are now being planned.

Recently, Middleton *et al*.^159^ published a phase I/II multicenter clinical trial of a bispecific fusion protein named tebentafusp that targets gp100, a melanocytic antigen that is expressed in both cutaneous and uveal melanoma. Tebentafusp can bind the gp100 protein presented on HLA-A*02 via its T cell receptor domain with a high affinity, while its anti-CD3 antibody domain binds surveilling CD3^+^ T cells to generate an immune response against the melanoma cells. The study’s metastatic uveal melanoma patients (19 out of 84 study patients) achieved a 1-year survival rate of 65%^159^. *In vitro* studies suggest that as few as 10 epitopes are needed to generate a sufficient immune response against melanoma cells, demonstrating tebentafusp’s high specificity for gp100 and its potential role in metastatic uveal melanoma^160,161^. A phase III clinical trial of tebentafusp will be completed in the near future.

In addition to immunotherapy, another therapeutic modality that is currently undergoing a phase Ib clinical trial is a nanoparticle therapy that uses photosensitive nanoparticles that preferentially bind tumor cells^162^. Once light-activated, these particles can selectively kill tumor cells with minimal damage to the surrounding normal tissues. This treatment modality, if proven successful in clinical trials, has the potential to preserve much of the patient’s vision and could be particularly groundbreaking in patients with small tumors that are close to critical ocular structures such as the optic nerve and the macula.

## Conclusion

The field of ocular oncology has seen major advancements in both diagnostic and treatment modalities for retinoblastoma and uveal melanoma in the last two decades. For retinoblastoma, the paradigm has shifted toward minimally invasive, targeted interventions, namely IAC and IVitC, which have dramatically turned retinoblastoma into a curable and manageable cancer in developed countries. On the other hand, innovations in the genomic analysis of uveal melanoma have allowed ocular oncologists to better characterize each tumor based on specific mutations and to predict the disease course and treatment outcomes. Although metastatic uveal melanoma remains a critical challenge for which no successful cure has been introduced, our conjoined efforts to detect and treat uveal melanoma at an early stage and to improve the treatment outcome of metastatic disease will hopefully come to fruition in the near future.

## References

[1] Broaddus E Topham A Singh AD: Incidence of retinoblastoma in the USA: 1975-2004. *Br J Ophthalmol.* 2009; 93(1): 21–3. 10.1136/bjo.2008.138750 18621794

[2] Singh AD Turell ME Topham AK: Uveal melanoma: Trends in incidence, treatment, and survival. *Ophthalmology.* 2011; 118(9): 1881–5. 10.1016/j.ophtha.2011.01.040 21704381

[3] Abramson DH Schefler AC: Update on retinoblastoma. *Retina.* 2004; 24(6): 828–48. 10.1097/00006982-200412000-00002 15579980

[4] American Academy of Pediatrics; Section on Ophthalmology; American Association for Pediatric Ophthalmology And Strabismus: Red reflex examination in neonates, infants, and children. *Pediatrics.* 2008; 122(6): 1401–4. 10.1542/peds.2008-2624 19047263

[5] Rushlow DE Mol BM Kennett JY: Characterisation of retinoblastomas without RB1 mutations: Genomic, gene expression, and clinical studies. *Lancet Oncol.* 2013; 14(4): 327–34. 10.1016/S1470-2045(13)70045-7 23498719

[6] Kleinerman RA Tucker MA Abramson DH: Risk of soft tissue sarcomas by individual subtype in survivors of hereditary retinoblastoma. *J Natl Cancer Inst.* 2007; 99(1): 24–31. 10.1093/jnci/djk002 17202110

[7] Marees T Moll AC Imhof SM: Risk of second malignancies in survivors of retinoblastoma: More than 40 years of follow-up. *J Natl Cancer Inst.* 2008; 100(24): 1771–9. 10.1093/jnci/djn394 19066271

[8] de Jong MC Kors WA de Graaf P: Trilateral retinoblastoma: A systematic review and meta-analysis. *Lancet Oncol.* 2014; 15(10): 1157–67. 10.1016/S1470-2045(14)70336-5 25126964

[9] Tonorezos ES Friedman DN Barnea D: Recommendations for Long-Term Follow-up of Adults with Heritable Retinoblastoma. *Ophthalmology.* 2020; 127(11): 1549–57. 10.1016/j.ophtha.2020.05.024 32422154 PMC7606265

[10] Fabian ID Abdallah E Abdullahi SU: Global Retinoblastoma Presentation and Analysis by National Income Level. *JAMA Oncol.* 2020; 6(5): 685–95. 10.1001/jamaoncol.2019.6716 32105305 PMC7047856

[11] Linn Murphree A: Intraocular retinoblastoma: The case for a new group classification. *Ophthalmol Clin North Am.* 2005; 18(1): 41–53, viii. 10.1016/j.ohc.2004.11.003 15763190

[12] Hamel P Heon E Gallie BL: Focal therapy in the management of retinoblastoma: When to start and when to stop. *J AAPOS.* 2000; 4(6): 334–7. 10.1067/mpa.2000.107902 11124666

[13] Shields CL de Potter P Himelstein BP: Chemoreduction in the initial management of intraocular retinoblastoma. *Arch Ophthalmol.* 1996; 114(11): 1330–8. 10.1001/archopht.1996.01100140530002 8906023

[14] Kingston JE Hungerford JL Madreperla SA: Results of combined chemotherapy and radiotherapy for advanced intraocular retinoblastoma. *Arch Ophthalmol.* 1996; 114(11): 1339–43. 10.1001/archopht.1996.01100140539004 8906024

[15] Shields CL Mashayekhi A Au AK: The International Classification of Retinoblastoma predicts chemoreduction success. *Ophthalmology.* 2006; 113(12): 2276–80. 10.1016/j.ophtha.2006.06.018 16996605

[16] Shields CL Jorge R Say EAT: Unilateral Retinoblastoma Managed With Intravenous Chemotherapy Versus Intra-Arterial Chemotherapy. Outcomes Based on the International Classification of Retinoblastoma. *Asia Pac J Ophthalmol (Phila).* 2016; 5(2): 97–103. 10.1097/APO.0000000000000172 26765038

[17] Ancona-Lezama D Dalvin LA Shields CL: Modern treatment of retinoblastoma: A 2020 review. *Indian J Ophthalmol.* 2020; 68(11): 2356–65. 10.4103/ijo.IJO_721_20 33120616 PMC7774148

[18] Shields CL Bas Z Tadepalli S: Long-term (20-year) real-world outcomes of intravenous chemotherapy (chemoreduction) for retinoblastoma in 964 eyes of 554 patients at a single centre. *Br J Ophthalmol.* 2020; 104(11): 1548–55. 10.1136/bjophthalmol-2019-315572 32051141

[19] Uusitalo MS van Quill KR Scott IU: Evaluation of chemoprophylaxis in patients with unilateral retinoblastoma with high-risk features on histopathologic examination. *Arch Ophthalmol.* 2001; 119(1): 41–8. 11146725

[20] Mendoza PR Grossniklaus HE: Therapeutic Options for Retinoblastoma. *Cancer Control.* 2016; 23(2): 99–109. 10.1177/107327481602300203 27218786

[21] Abramson DH Daniels AB Marr BP: Intra-Arterial Chemotherapy (Ophthalmic Artery Chemosurgery) for Group D Retinoblastoma. *PLoS One.* 2016; 11(1): e0146582. 10.1371/journal.pone.0146582 26756643 PMC4710506

[22] Santapuram PR Schremp EA Friedman DL: Adverse Events, Treatment Burden, and Outcomes of Intravenous versus Intra-arterial Chemotherapy for Retinoblastoma. *Ophthalmol Retina.* 2021; 5(3): 309–312. 10.1016/j.oret.2020.09.006 32920208

[23] Chen Q Zhang B Dong Y: Comparison between intravenous chemotherapy and intra-arterial chemotherapy for retinoblastoma: A meta-analysis. *BMC Cancer.* 2018; 18(1): 486. 10.1186/s12885-018-4406-6 29703164 PMC5924469

[24] Francis JH Levin AM Zabor EC: Ten-year experience with ophthalmic artery chemosurgery: Ocular and recurrence-free survival. *PLoS One.* 2018; 13(5): e0197081. 10.1371/journal.pone.0197081 29791475 PMC5965845

[25] Abramson DH Fabius AWM Issa R: Advanced Unilateral Retinoblastoma: The Impact of Ophthalmic Artery Chemosurgery on Enucleation Rate and Patient Survival at MSKCC. *PLoS One.* 2015; 10(12): e0145436. 10.1371/journal.pone.0145436 26709699 PMC4692433

[26] Abramson DH Fabius AWM Francis JH: Ophthalmic artery chemosurgery for eyes with advanced retinoblastoma. *Ophthalmic Genet.* 2017; 38(1): 16–21. 10.1080/13816810.2016.1244695 28095092 PMC5475401

[27] Shields CL Manjandavida FP Lally SE: Intra-arterial chemotherapy for retinoblastoma in 70 eyes: Outcomes based on the international classification of retinoblastoma. *Ophthalmology.* 2014; 121(7): 1453–60. 10.1016/j.ophtha.2014.01.026 24656794

[28] Chen M Jiang H Zhang J: Outcome of intra-arterial chemotherapy for retinoblastoma and its influencing factors: A retrospective study. *Acta Ophthalmol.* 2017; 95(6): 613–8. 10.1111/aos.13333 27874261

[29] Munier FL Mosimann P Puccinelli F: First-line intra-arterial versus intravenous chemotherapy in unilateral sporadic group D retinoblastoma: Evidence of better visual outcomes, ocular survival and shorter time to success with intra-arterial delivery from retrospective review of 20 years of treatment. *Br J Ophthalmol.* 2017; 101(8): 1086–93. 10.1136/bjophthalmol-2016-309298 27927678 PMC5537510

[30] Francis JH Roosipu N Levin AM: Current Treatment of Bilateral Retinoblastoma: The Impact of Intraarterial and Intravitreous Chemotherapy. *Neoplasia.* 2018; 20(8): 757–63. 10.1016/j.neo.2018.05.007 29940303 PMC6020084

[31] Francis JH Abramson DH Gobin YP: Efficacy and toxicity of second-course ophthalmic artery chemosurgery for retinoblastoma. *Ophthalmology.* 2015; 122(5): 1016–22. 10.1016/j.ophtha.2014.11.029 25616769 PMC4994525

[32] Gobin YP Dunkel IJ Marr BP: Intra-arterial chemotherapy for the management of retinoblastoma: Four-year experience. *Arch Ophthalmol.* 2011; 129(6): 732–7. 10.1001/archophthalmol.2011.5 21320950

[33] Gobin YP Dunkel IJ Marr BP: Combined, sequential intravenous and intra-arterial chemotherapy (bridge chemotherapy) for young infants with retinoblastoma. *PLoS One.* 2012; 7(9): e44322. 10.1371/journal.pone.0044322 23028521 PMC3445577

[34] Sweid A Hammoud B Weinberg JH: Intra-Arterial Chemotherapy for Retinoblastoma in Infants ≤10 kg: 74 Treated Eyes with 222 IAC Sessions. *AJNR Am J Neuroradiol.* 2020; 41(7): 1286–92. 10.3174/ajnr.A6590 32586963 PMC7357663

[35] Chen Q Zhang B Dong Y: Intra-arterial chemotherapy as primary or secondary treatment for infants diagnosed with advanced retinoblastoma before 3 months of age. *BMC Cancer.* 2019; 19(1): 693. 10.1186/s12885-019-5844-5 31307410 PMC6631809

[36] Manjandavida FP Xia J Zhang J: In-utero ultrasonography detection of fetal retinoblastoma and neonatal selective ophthalmic artery chemotherapy. *Indian J Ophthalmol.* 2019; 67(6): 958–60. 10.4103/ijo.IJO_340_19 31124531 PMC6552617

[37] Magan T Khoo CTL Jabbour PM: Intraarterial chemotherapy for retinoblastoma in a 2-month-old infant. *Retin Cases Brief Rep.* 2017; 11(1): 24–6. 10.1097/ICB.0000000000000279 26756523

[38] Kim RS Dannenbaum MJ Lin MW: Use of femoral artery ultrasound during intraarterial chemotherapy for children under 10 kg with retinoblastoma. *Retina.* 2018; 38(7): 1420–6. 10.1097/IAE.0000000000001713 28541962

[39] Shields CL Kaliki S Shah SU: Minimal exposure (one or two cycles) of intra-arterial chemotherapy in the management of retinoblastoma. *Ophthalmology.* 2012; 119(1): 188–92. 10.1016/j.ophtha.2011.06.036 21975042

[40] Ghassemi F Shields CL: Intravitreal melphalan for refractory or recurrent vitreous seeding from retinoblastoma. *Arch Ophthalmol.* 2012; 130(10): 1268–71. 10.1001/archophthalmol.2012.1983 23044940

[41] Munier FL Gaillard MC Balmer A: Intravitreal chemotherapy for vitreous disease in retinoblastoma revisited: From prohibition to conditional indications. *Br J Ophthalmol.* 2012; 96(8): 1078–83. 10.1136/bjophthalmol-2011-301450 22694968

[42] Munier FL Beck-Popovic M Chantada GL: Conservative management of retinoblastoma: Challenging orthodoxy without compromising the state of metastatic grace. "Alive, with good vision and no comorbidity". *Prog Retin Eye Res.* 2019; 73: 100764. 10.1016/j.preteyeres.2019.05.005 31173880

[43] Munier FL Soliman S Moulin AP: Profiling safety of intravitreal injections for retinoblastoma using an anti-reflux procedure and sterilisation of the needle track. *Br J Ophthalmol.* 2012; 96(8): 1084–7. 10.1136/bjophthalmol-2011-301016 22368262

[44] Munier FL: Classification and management of seeds in retinoblastoma. Ellsworth Lecture Ghent August 24th 2013. *Ophthalmic Genet.* 2014; 35(4): 193–207. 10.3109/13816810.2014.973045 25321846 PMC4245997

[45] Munier FL Gaillard MC Decembrini S: Intracameral Chemotherapy (Melphalan) for Aqueous Seeding in Retinoblastoma: Bicameral Injection Technique and Related Toxicity in a Pilot Case Study. *Ocul Oncol Pathol.* 2017; 3(2): 149–55. 10.1159/000453617 28868287 PMC5566720

[46] Munier FL Moulin A Gaillard MC: Intracameral Chemotherapy for Globe Salvage in Retinoblastoma with Secondary Anterior Chamber Invasion. *Ophthalmology.* 2018; 125(4): 615–7. 10.1016/j.ophtha.2017.11.010 29208450

[47] Cassoux N Aerts I Lumbroso-Le Rouic L: Eye Salvage with Combination of Intravitreal and Intracameral Melphalan Injection for Recurrent Retinoblastoma with Anterior Chamber Involvement: Report of a Case. *Ocul Oncol Pathol.* 2017; 3(2): 129–32. 10.1159/000452305 28868284 PMC5566760

[48] Shields CL Mashayekhi A Demirci H: Practical approach to management of retinoblastoma. *Arch Ophthalmol.* 2004; 122(5): 729–35. 10.1001/archopht.122.5.729 15136321

[49] Schefler AC Kim RS: Recent advancements in the management of retinoblastoma and uveal melanoma [version 1; peer review: 2 approved]. *F1000Res.* 2018; 7: F1000 Faculty Rev-476. 10.12688/f1000research.11941.1 29755733 PMC5911936

[50] Singh G Daniels AB: Disparities in Retinoblastoma Presentation, Treatment, and Outcomes in Developed and Less-Developed Countries. *Semin Ophthalmol.* 2016; 31(4): 310–6. 10.3109/08820538.2016.1154177 27127937

[51] Scelfo C Francis JH Khetan V: An international survey of classification and treatment choices for group D retinoblastoma. *Int J Ophthalmol.* 2017; 10(6): 961–7. 10.18240/ijo.2017.06.20 28730089 PMC5515152

[52] Fabian ID Stacey AW Johnson KC: Primary enucleation for group D retinoblastoma in the era of systemic and targeted chemotherapy: The price of retaining an eye. *Br J Ophthalmol.* 2018; 102(2): 265–71. 10.1136/bjophthalmol-2017-310624 28659391

[53] Chantada GL Qaddoumi I Canturk S: Strategies to manage retinoblastoma in developing countries. *Pediatr Blood Cancer.* 2011; 56(3): 341–8. 10.1002/pbc.22843 21225909

[54] Abramson DH Ellsworth RM Grumbach N: Retinoblastoma: Correlation between age at diagnosis and survival. *J Pediatr Ophthalmol Strabismus.* 1986; 23(4): 174–7. 3746592 10.3928/0191-3913-19860701-06

[55] Rodrigues KES Latorre MdRDO de Camargo B: Delayed diagnosis in retinoblastoma. *J Pediatr (Rio J).* 2004; 80(6): 511–6. 10.2223/1266 15622429

[56] Leal-Leal C Flores-Rojo M Medina-Sansón A: A multicentre report from the Mexican Retinoblastoma Group. *Br J Ophthalmol.* 2004; 88(8): 1074–7. 10.1136/bjo.2003.035642 15258028 PMC1772266

[57] Canturk S Qaddoumi I Khetan V: Survival of retinoblastoma in less-developed countries impact of socioeconomic and health-related indicators. *Br J Ophthalmol.* 2010; 94(11): 1432–6. 10.1136/bjo.2009.168062 20733021

[58] Bowman RJC Mafwiri M Luthert P: Outcome of retinoblastoma in east Africa. *Pediatr Blood Cancer.* 2008; 50(1): 160–2. 10.1002/pbc.21080 17120241

[59] Reddy MA Butt M Hinds A-M: Prognostic Information for Known Genetic Carriers of RB1 Pathogenic Variants (Germline and Mosaic). *Ophthalmol Retina.* 2021; 5(4): 381–7. 10.1016/j.oret.2020.08.010 32835838

[60] Pradhan MA Ng Y Strickland A: Role of genetic testing in retinoblastoma management at a tertiary referral centre. *Clin Exp Ophthalmol.* 2010; 38(3): 231–6. 10.1111/j.1442-9071.2010.02239.x 20447117

[61] Gerrish A Stone E Clokie S: Non-invasive diagnosis of retinoblastoma using cell-free DNA from aqueous humour. *Br J Ophthalmol.* 2019; 103(5): 721–724. 10.1136/bjophthalmol-2018-313005 30745306 PMC6709774

[62] Berry JL Xu L Murphree AL: Potential of Aqueous Humor as a Surrogate Tumor Biopsy for Retinoblastoma. *JAMA Ophthalmol.* 2017; 135(11): 1221–30. 10.1001/jamaophthalmol.2017.4097 29049475 PMC5710399

[63] Berry JL Xu L Kooi I: Genomic cfDNA Analysis of Aqueous Humor in Retinoblastoma Predicts Eye Salvage: The Surrogate Tumor Biopsy for Retinoblastoma. *Mol Cancer Res.* 2018; 16(11): 1701–1712. 10.1158/1541-7786.MCR-18-0369 30061186 PMC6214755

[64] Wang H Bauzon F Ji P: Skp2 is required for survival of aberrantly proliferating *Rb1*-deficient cells and for tumorigenesis in *Rb1*^+/-^ mice. *Nat Genet.* 2010; 42(1): 83–8. 10.1038/ng.498 19966802 PMC2990528

[65] Lee TC Almeida D Claros N: Cell cycle-specific and cell type-specific expression of Rb in the developing human retina. *Invest Ophthalmol Vis Sci.* 2006; 47(12): 5590–5598. 10.1167/iovs.06-0063 17122153

[66] Xu XL Fang Y Lee TC: Retinoblastoma has properties of a cone precursor tumor and depends upon cone-specific MDM2 signaling. *Cell.* 2009; 137(6): 1018–31. 10.1016/j.cell.2009.03.051 19524506 PMC5659855

[67] Xu XL Singh HP Wang L: Rb suppresses human cone-precursor-derived retinoblastoma tumours. *Nature.* 2014; 514(7522): 385–8. 10.1038/nature13813 25252974 PMC4232224

[68] Chen X Kunda PE Lin J: SYK-targeted dendritic cell-mediated cytotoxic T lymphocytes enhance the effect of immunotherapy on retinoblastoma. *J Cancer Res Clin Oncol.* 2018; 144(4): 675–84. 10.1007/s00432-018-2584-x 29372378 PMC5843685

[69] Li J Di C Jing J: OTX2 is a therapeutic target for retinoblastoma and may function as a common factor between C-MYC, CRX, and phosphorylated RB pathways. *Int J Oncol.* 2015; 47(5): 1703–10. 10.3892/ijo.2015.3179 26397460

[70] Yu MD Masoomian B Shields JA: *BAP1* Germline Mutation Associated with Bilateral Primary Uveal Melanoma. *Ocul Oncol Pathol.* 2020; 6(1): 10–14. 10.1159/000499570 32002398 PMC6984137

[71] Shields CL Kaliki S Shah SU: Iris melanoma: Features and prognosis in 317 children and adults. *J AAPOS.* 2012; 16(1): 10–6. 10.1016/j.jaapos.2011.10.012 22370659

[72] Singh AD Topham A: Survival rates with uveal melanoma in the United States: 1973–1997. *Ophthalmology.* 2003; 110(5): 962–5. 10.1016/S0161-6420(03)00077-0 12750098

[73] Diener-West M Earle JD Fine SL: The COMS randomized trial of iodine 125 brachytherapy for choroidal melanoma, III: Initial mortality findings. COMS Report No. 18. *Arch Ophthalmol.* 2001; 119(7): 969–82. 10.1001/archopht.119.7.969 11448319

[74] Kujala E Mäkitie T Kivelä T: Very long-term prognosis of patients with malignant uveal melanoma. *Invest Ophthalmol Vis Sci.* 2003; 44(11): 4651–9. 10.1167/iovs.03-0538 14578381

[75] Eskelin S Pyrhönen S Summanen P: Tumor doubling times in metastatic malignant melanoma of the uvea: Tumor progression before and after treatment. *Ophthalmology.* 2000; 107(8): 1443–9. 10.1016/s0161-6420(00)00182-2 10919885

[76] Collaborative Ocular Melanoma Study Group: Assessment of metastatic disease status at death in 435 patients with large choroidal melanoma in the Collaborative Ocular Melanoma Study (COMS): COMS report no. 15. *Arch Ophthalmol.* 2001; 119(5): 670–6. 10.1001/archopht.119.5.670 11346394

[77] Diener-West M Reynolds SM Agugliaro DJ: Development of metastatic disease after enrollment in the COMS trials for treatment of choroidal melanoma: Collaborative Ocular Melanoma Study Group Report No. 26. *Arch Ophthalmol.* 2005; 123(12): 1639–43. 10.1001/archopht.123.12.1639 16344433

[78] Carvajal RD Schwartz GK Tezel T: Metastatic disease from uveal melanoma: Treatment options and future prospects. *Br J Ophthalmol.* 2017; 101(1): 38–44. 10.1136/bjophthalmol-2016-309034 27574175 PMC5256122

[79] Lane AM Kim IK Gragoudas ES: Survival Rates in Patients After Treatment for Metastasis From Uveal Melanoma. *JAMA Ophthalmol.* 2018; 136(9): 981–6. 10.1001/jamaophthalmol.2018.2466 29955797 PMC6142974

[80] Qiu M Shields CL: Choroidal Nevus in the United States Adult Population: Racial Disparities and Associated Factors in the National Health and Nutrition Examination Survey. *Ophthalmology.* 2015; 122(10): 2071–83. 10.1016/j.ophtha.2015.06.008 26255109

[81] Singh AD Kalyani P Topham A: Estimating the risk of malignant transformation of a choroidal nevus. *Ophthalmology.* 2005; 112(10): 1784–9. 10.1016/j.ophtha.2005.06.011 16154197

[82] Rimoldi D Salvi S Liénard D: Lack of BRAF mutations in uveal melanoma. *Cancer Res.* 2003; 63(18): 5712–5. 14522889

[83] Yoo JH Shi DS Grossmann AH: ARF6 Is an Actionable Node that Orchestrates Oncogenic GNAQ Signaling in Uveal Melanoma. *Cancer Cell.* 2016; 29(6): 889–904. 10.1016/j.ccell.2016.04.015 27265506 PMC5027844

[84] Carbone M Yang H Pass HI: BAP1 and cancer. *Nat Rev Cancer.* 2013; 13(3): 153–9. 10.1038/nrc3459 23550303 PMC3792854

[85] Bononi A Giorgi C Patergnani S: BAP1 regulates IP3R3-mediated Ca^2+^ flux to mitochondria suppressing cell transformation. *Nature.* 2017; 546(7659): 549–53. 10.1038/nature22798 28614305 PMC5581194

[86] Matatall KA Agapova OA Onken MD: BAP1 deficiency causes loss of melanocytic cell identity in uveal melanoma. *BMC Cancer.* 2013; 13: 371. 10.1186/1471-2407-13-371 23915344 PMC3846494

[87] Smit KN Jager MJ de Klein A: Uveal melanoma: Towards a molecular understanding. *Prog Retin Eye Res.* 2020; 75: 100800. 10.1016/j.preteyeres.2019.100800 31563544

[88] Jager MJ Shields CL Cebulla CM: Uveal melanoma. *Nat Rev Dis Primers.* 2020; 6(1): 24. 10.1038/s41572-020-0158-0 32273508

[89] Furney SJ Pedersen M Gentien D: *SF3B1* mutations are associated with alternative splicing in uveal melanoma. *Cancer Discov.* 2013; 3(10): 1122–9. 10.1158/2159-8290.CD-13-0330 23861464 PMC5321577

[90] Darman RB Seiler M Agrawal AA: Cancer-Associated SF3B1 Hotspot Mutations Induce Cryptic 3' Splice Site Selection through Use of a Different Branch Point. *Cell Rep.* 2015; 13(5): 1033–45. 10.1016/j.celrep.2015.09.053 26565915

[91] Martin M Maßhöfer L Temming P: Exome sequencing identifies recurrent somatic mutations in *EIF1AX* and *SF3B1* in uveal melanoma with disomy 3. *Nat Genet.* 2013; 45(8): 933–6. 10.1038/ng.2674 23793026 PMC4307600

[92] Onken MD Worley LA Ehlers JP: Gene expression profiling in uveal melanoma reveals two molecular classes and predicts metastatic death. *Cancer Res.* 2004; 64(20): 7205–9. 10.1158/0008-5472.CAN-04-1750 15492234 PMC5407684

[93] Harbour JW: A prognostic test to predict the risk of metastasis in uveal melanoma based on a 15-gene expression profile. *Methods Mol Biol.* 2014; 1102: 427–40. 10.1007/978-1-62703-727-3_22 24258991 PMC4476294

[94] Field MG Harbour JW: Recent developments in prognostic and predictive testing in uveal melanoma. *Curr Opin Ophthalmol.* 2014; 25(3): 234–9. 10.1097/ICU.0000000000000051 24713608 PMC4467564

[95] Shields CL Say EAT Hasanreisoglu M: Personalized Prognosis of Uveal Melanoma Based on Cytogenetic Profile in 1059 Patients over an 8-Year Period: The 2017 Harry S. Gradle Lecture. *Ophthalmology.* 2017; 124(10): 1523–31. 10.1016/j.ophtha.2017.04.003 28495150

[96] Yavuzyigitoglu S Koopmans AE Verdijk RM: Uveal Melanomas with SF3B1 Mutations: A Distinct Subclass Associated with Late-Onset Metastases. *Ophthalmology.* 2016; 123(5): 1118–28. 10.1016/j.ophtha.2016.01.023 26923342

[97] Shain AH Bagger MM Yu R: The genetic evolution of metastatic uveal melanoma. *Nat Genet.* 2019; 51(7): 1123–30. 10.1038/s41588-019-0440-9 31253977 PMC6632071

[98] Rodrigues M Mobuchon L Houy A: Evolutionary Routes in Metastatic Uveal Melanomas Depend on *MBD4* Alterations. *Clin Cancer Res.* 2019; 25: 5513–24. 10.1158/1078-0432.CCR-19-1215 31227496

[99] Piaggio F Tozzo V Bernardi C: Secondary Somatic Mutations in G-Protein-Related Pathways and Mutation Signatures in Uveal Melanoma. *Cancers (Basel).* 2019; 11(11): 1688. 10.3390/cancers11111688 31671564 PMC6896012

[100] Jampol LM Moy CS Murray TG: The COMS randomized trial of iodine 125 brachytherapy for choroidal melanoma: IV. Local treatment failure and enucleation in the first 5 years after brachytherapy. COMS report no. 19. *Ophthalmology.* 2002; 109(12): 2197–206. 10.1016/s0161-6420(02)01277-0 12466159

[101] Nag S Quivey JM Earle JD: The American Brachytherapy Society recommendations for brachytherapy of uveal melanomas. *Int J Radiat Oncol Biol Phys.* 2003; 56(2): 544–55. 10.1016/s0360-3016(03)00006-3 12738332

[102] Takiar V Voong KR Gombos DS: A choice of radionuclide: Comparative outcomes and toxicity of ruthenium-106 and iodine-125 in the definitive treatment of uveal melanoma. *Pract Radiat Oncol.* 2015; 5(3): e169–e176. 10.1016/j.prro.2014.09.005 25423888

[103] Wilkinson DA Kolar M Fleming PA: Dosimetric comparison of 106Ru and 125I plaques for treatment of shallow (<or=5 mm) choroidal melanoma lesions. *Br J Radiol.* 2008; 81(970): 784–9. 10.1259/bjr/76813976 18628320

[104] Damato B Kacperek A Errington D: Proton beam radiotherapy of uveal melanoma. *Saudi J Ophthalmol.* 2013; 27(3): 151–7. 10.1016/j.sjopt.2013.06.014 24227980 PMC3770228

[105] Papakostas TD Lane AM Morrison M: Long-term Outcomes After Proton Beam Irradiation in Patients With Large Choroidal Melanomas. *JAMA Ophthalmol.* 2017; 135(11): 1191–6. 10.1001/jamaophthalmol.2017.3805 29049518 PMC5710395

[106] Wang Z Nabhan M Schild SE: Charged particle radiation therapy for uveal melanoma: A systematic review and meta-analysis. *Int J Radiat Oncol Biol Phys.* 2013; 86(1): 18–26. 10.1016/j.ijrobp.2012.08.026 23040219

[107] Mishra KK Daftari IK: Proton therapy for the management of uveal melanoma and other ocular tumors. *Chin Clin Oncol.* 2016; 5(4): 50. 10.21037/cco.2016.07.06 27558251

[108] Bellerive C Aziz HA Bena J: Local Failure After Episcleral Brachytherapy for Posterior Uveal Melanoma: Patterns, Risk Factors, and Management. *Am J Ophthalmol.* 2017; 177: 9–16. 10.1016/j.ajo.2017.01.024 28163118

[109] Le BHA Kim JW Deng H: Outcomes of choroidal melanomas treated with eye physics plaques: A 25-year review. *Brachytherapy.* 2018; 17(6): 981–9. 10.1016/j.brachy.2018.07.002 30082188 PMC6613587

[110] Tann AW Teh BS Scarboro SB: Early outcomes of uveal melanoma treated with intraoperative ultrasound guided brachytherapy using custom built plaques. *Pract Radiat Oncol.* 2017; 7(4): e275–e282. 10.1016/j.prro.2017.01.002 28377140

[111] Ophthalmic Oncology Task Force: Local Recurrence Significantly Increases the Risk of Metastatic Uveal Melanoma. *Ophthalmology.* 2016; 123(1): 86–91. 10.1016/j.ophtha.2015.09.014 26505803

[112] Seibel I Cordini D Rehak M: Local Recurrence After Primary Proton Beam Therapy in Uveal Melanoma: Risk Factors, Retreatment Approaches, and Outcome. *Am J Ophthalmol.* 2015; 160(4): 628–36. 10.1016/j.ajo.2015.06.017 26133249

[113] Collaborative Ocular Melanoma Study Group: The COMS randomized trial of iodine 125 brachytherapy for choroidal melanoma: V. Twelve-year mortality rates and prognostic factors: COMS report No. 28. *Arch Ophthalmol.* 2006; 124(12): 1684–93. 10.1001/archopht.124.12.1684 17159027

[114] Harbour JW Char DH Kroll S: Metastatic Risk for Distinct Patterns of Postirradiation Local Recurrence of Posterior Uveal Melanoma. *Ophthalmology.* 1997; 104(11): 1785–93; discussion 1792–3. 10.1016/s0161-6420(97)30025-6 9373108

[115] Riechardt AI Cordini D Dobner B: Salvage proton beam therapy in local recurrent uveal melanoma. *Am J Ophthalmol.* 2014; 158(5): 948–56. 10.1016/j.ajo.2014.07.013 25038327

[116] Astrahan MA Luxton G Jozsef G: An interactive treatment planning system for ophthalmic plaque radiotherapy. *Int J Radiat Oncol Biol Phys.* 1990; 18(3): 679–87. 10.1016/0360-3016(90)90077-w 2318702

[117] Harbour JW Murray TG Byrne SF: Intraoperative echographic localization of iodine 125 episcleral radioactive plaques for posterior uveal melanoma. *Retina.* 1996; 16(2): 129–34. 10.1097/00006982-199616020-00008 8724957

[118] Tabandeh H Chaudhry NA Murray TG: Intraoperative echographic localization of iodine-125 episcleral plaque for brachytherapy of choroidal melanoma. *Am J Ophthalmol.* 2000; 129(2): 199–204. 10.1016/s0002-9394(99)00315-3 10682973

[119] Finger PT: Intraoperative echographic localization of iodine-125 episcleral plaque for brachytherapy of choroidal melanoma. *Am J Ophthalmol.* 2000; 130(4): 539–40. 10.1016/s0002-9394(00)00549-3 11183561

[120] Aziz HA Al Zahrani YA Bena J: Episcleral brachytherapy of uveal melanoma: Role of intraoperative echographic confirmation. *Br J Ophthalmol.* 2017; 101(6): 747–51. 10.1136/bjophthalmol-2016-309153 27574179

[121] Wisely CE Hadziahmetovic M Reem RE: Long-term visual acuity outcomes in patients with uveal melanoma treated with 125I episcleral OSU-Nag plaque brachytherapy. *Brachytherapy.* 2016; 15(1): 12–22. 10.1016/j.brachy.2015.09.013 26525215 PMC4990815

[122] Astrahan MA Luxton G Pu Q: Conformal episcleral plaque therapy. *Int J Radiat Oncol Biol Phys.* 1997; 39(2): 505–19. 10.1016/s0360-3016(97)00118-1 9308957

[123] Astrahan MA Luxton G Jozsef G: Optimization of 125I ophthalmic plaque brachytherapy. *Med Phys.* 1990; 17(6): 1053–7. 10.1118/1.596585 2280735

[124] Gragoudas ES: Proton beam irradiation of uveal melanomas: The first 30 years. The Weisenfeld Lecture. *Invest Ophthalmol Vis Sci.* 2006; 47(11): 4666–73. 10.1167/iovs.06-0659 17065472

[125] Irvine AR Wood IS: Radiation Retinopathy as an Experimental Model for Ischemic Proliferative Retinopathy and Rubeosis Iridis. *Am J Ophthalmol.* 1987; 103(6): 790–7. 10.1016/s0002-9394(14)74395-8 2438938

[126] Gündüz K Shields CL Shields JA: Radiation retinopathy following plaque radiotherapy for posterior uveal melanoma. *Arch Ophthalmol.* 1999; 117(5): 609–14. 10.1001/archopht.117.5.609 10326957

[127] Horgan N Shields CL Mashayekhi A: Early macular morphological changes following plaque radiotherapy for uveal melanoma. *Retina.* 2008; 28(2): 263–73. 10.1097/IAE.0b013e31814b1b75 18301032

[128] Shah NV Houston SK Markoe AM: Early SD-OCT diagnosis followed by prompt treatment of radiation maculopathy using intravitreal bevacizumab maintains functional visual acuity. *Clin Ophthalmol.* 2012; 6: 1739–48. 10.2147/OPTH.S34949 23152651 PMC3497449

[129] Mashayekhi A Rojanaporn D Al-Dahmash S: Monthly intravitreal bevacizumab for macular edema after iodine-125 plaque radiotherapy of uveal melanoma. *Eur J Ophthalmol.* 2014; 24(2): 228–34. 10.5301/ejo.5000352 23934823

[130] Horgan N Shields CL Mashayekhi A: Periocular triamcinolone for prevention of macular edema after iodine 125 plaque radiotherapy of uveal melanoma. *Retina.* 2008; 28(7): 987–95. 10.1097/IAE.0b013e31816b3192 18698302

[131] Shields CL Demirci H Dai V: Intravitreal triamcinolone acetonide for radiation maculopathy after plaque radiotherapy for choroidal melanoma. *Retina.* 2005; 25(7): 868–74. 10.1097/00006982-200510000-00009 16205566

[132] Baillif S Maschi C Gastaud P: Intravitreal dexamethasone 0.7-mg implant for radiation macular edema after proton beam therapy for choroidal melanoma. *Retina.* 2013; 33(9): 1784–90. 10.1097/IAE.0b013e31829234fa 23652581

[133] Frizziero L Parrozzani R Trainiti S: Intravitreal dexamethasone implant in radiation-induced macular oedema. *Br J Ophthalmol.* 2017; 101(12): 1699–703. 10.1136/bjophthalmol-2017-310220 28404670

[134] Murray TG Latiff A Villegas VM: Aflibercept for Radiation Maculopathy Study: A Prospective, Randomized Clinical Study. *Ophthalmol Retina.* 2019; 3(7): 561–6. 10.1016/j.oret.2019.02.009 31277797

[135] Fallico M Reibaldi M Avitabile T: Intravitreal aflibercept for the treatment of radiation-induced macular edema after ruthenium 106 plaque radiotherapy for choroidal melanoma. *Graefes Arch Clin Exp Ophthalmol.* 2019; 257(7): 1547–54. 10.1007/s00417-019-04347-6 31081526

[136] Schefler AC Fuller D Anand R: Randomized Trial of Monthly Versus As-Needed Intravitreal Ranibizumab for Radiation Retinopathy-Related Macular Edema: 1-Year Outcomes. *Am J Ophthalmol.* 2020; 216: 165–73. 10.1016/j.ajo.2020.03.045 32278771

[137] Melia BM Abramson DH Albert DM: Collaborative ocular melanoma study (COMS) randomized trial of I-125 brachytherapy for medium choroidal melanoma. I. Visual acuity after 3 years COMS report no. 16. *Ophthalmology.* 2001; 108(2): 348–66. 10.1016/s0161-6420(00)00526-1 11158813

[138] Seibel I Vollhardt D Riechardt AI: Influence of Ranibizumab versus laser photocoagulation on radiation retinopathy (RadiRet) - a prospective randomized controlled trial. *Graefes Arch Clin Exp Ophthalmol.* 2020; 258(4): 869–78. 10.1007/s00417-020-04618-7 32112140 PMC7575494

[139] Kim IK Lane AM Jain P: Ranibizumab for the Prevention of Radiation Complications in Patients Treated With Proton Beam Irradiation for Choroidal Melanoma. *Trans Am Ophthalmol Soc.* 2016; 114: T2. 27630373 PMC5012854

[140] Shah SU Shields CL Bianciotto CG: Intravitreal bevacizumab at 4-month intervals for prevention of macular edema after plaque radiotherapy of uveal melanoma. *Ophthalmology.* 2014; 121(1): 269–75. 10.1016/j.ophtha.2013.08.039 24139123

[141] Corrêa ZM Augsburger JJ: Independent Prognostic Significance of Gene Expression Profile Class and Largest Basal Diameter of Posterior Uveal Melanomas. *Am J Ophthalmol.* 2016; 162: 20–27.e1. 10.1016/j.ajo.2015.11.019 26596399

[142] Demirci H Niziol LM Ozkurt Z: Do Largest Basal Tumor Diameter and the American Joint Committee on Cancer's Cancer Staging Influence Prognostication by Gene Expression Profiling in Choroidal Melanoma. *Am J Ophthalmol.* 2018; 195: 83–92. 10.1016/j.ajo.2018.07.033 30081017

[143] Binkley EM Bena JF Davanzo JM: Gene Expression Profiling Prognostication of Posterior Uveal Melanoma: Does Size Matter? *Ophthalmol Retina.* 2020; 4(6): 620–9. 10.1016/j.oret.2019.12.020 32081600

[144] Berry DE Schefler AC Seider MI: Correlation of gene expression profile status and american joint commission on cancer stage in uveal melanoma. *Retina.* 2020; 40(2): 214–24. 10.1097/IAE.0000000000002385 31972790 PMC6506408

[145] Walter SD Chao DL Feuer W: Prognostic Implications of Tumor Diameter in Association With Gene Expression Profile for Uveal Melanoma. *JAMA Ophthalmol.* 2016; 134(7): 734–40. 10.1001/jamaophthalmol.2016.0913 27123792 PMC4966166

[146] Ikeda H Lethé B Lehmann F: Characterization of an Antigen That Is Recognized on a Melanoma Showing Partial HLA Loss by CTL Expressing an NK Inhibitory Receptor. *Immunity.* 1997; 6(2): 199–208. 10.1016/s1074-7613(00)80426-4 9047241

[147] Epping MT Wang L Edel MJ: The human tumor antigen PRAME is a dominant repressor of retinoic acid receptor signaling. *Cell.* 2005; 122(6): 835–47. 10.1016/j.cell.2005.07.003 16179254

[148] Field MG Durante MA Decatur CL: Epigenetic reprogramming and aberrant expression of PRAME are associated with increased metastatic risk in Class 1 and Class 2 uveal melanomas. *Oncotarget.* 2016; 7(37): 59209–19. 10.18632/oncotarget.10962 27486988 PMC5312306

[149] Field MG Decatur CL Kurtenbach S: PRAME as an Independent Biomarker for Metastasis in Uveal Melanoma. *Clin Cancer Res.* 2016; 22(5): 1234–42. 10.1158/1078-0432.CCR-15-2071 26933176 PMC4780366

[150] Cai L Paez-Escamilla M Walter SD: Gene Expression Profiling and *PRAME* Status Versus Tumor-Node-Metastasis Staging for Prognostication in Uveal Melanoma. *Am J Ophthalmol.* 2018; 195: 154–60. 10.1016/j.ajo.2018.07.045 30092184 PMC6214741

[151] Schefler AC Koca E Bernicker EH: Relationship between clinical features, GEP class, and *PRAME* expression in uveal melanoma. *Graefes Arch Clin Exp Ophthalmol.* 2019; 257(7): 1541–5. 10.1007/s00417-019-04335-w 31065847

[152] Gupta K McCannel CA Kamrava M: Tumor-height regression rate after brachytherapy between choroidal melanoma gene expression profile classes: Effect of controlling for tumor height. *Graefes Arch Clin Exp Ophthalmol.* 2016; 254(7): 1371–8. 10.1007/s00417-016-3305-2 26907932

[153] Corrêa ZM Augsburger JJ: Relationship between rate of posterior uveal melanoma flattening following plaque radiotherapy and gene expression profile class of tumor cells. *Invest Ophthalmol Vis Sci.* 2014; 55(1): 556–9. 10.1167/iovs.13-13381 24408979

[154] Rao RC Khan M Badiyan SN: Gene expression profiling and regression rate of irradiated uveal melanomas. *Ophthalmic Surg Lasers Imaging Retina.* 2015; 46(3): 333–7. 10.3928/23258160-20150323-06 25856819 PMC4398963

[155] Mruthyunjaya P Seider MI Stinnett S: Association between Tumor Regression Rate and Gene Expression Profile after Iodine 125 Plaque Radiotherapy for Uveal Melanoma. *Ophthalmology.* 2017; 124(10): 1532–9.. 10.1016/j.ophtha.2017.04.013 28549517

[156] Liu TYA Zhu H Chen H: Gene Expression Profile Prediction in Uveal Melanoma Using Deep Learning: A Pilot Study for the Development of an Alternative Survival Prediction Tool. *Ophthalmol Retina.* 2020; 4(12): 1213–5. 10.1016/j.oret.2020.06.023 32565384

[157] Durante MA Rodriguez DA Kurtenbach S: Single-cell analysis reveals new evolutionary complexity in uveal melanoma. *Nat Commun.* 2020; 11(1): 496. 10.1038/s41467-019-14256-1 31980621 PMC6981133

[158] Faião-Flores F Emmons MF Durante MA: HDAC Inhibition Enhances the *In Vivo* Efficacy of MEK Inhibitor Therapy in Uveal Melanoma. *Clin Cancer Res.* 2019; 25(18): 5686–701. 10.1158/1078-0432.CCR-18-3382 31227503 PMC6744978

[159] Middleton MR McAlpine C Woodcock VK: Tebentafusp, A TCR/Anti-CD3 Bispecific Fusion Protein Targeting gp100, Potently Activated Antitumor Immune Responses in Patients with Metastatic Melanoma. *Clin Cancer Res.* 2020; 26(22): 5869–78. 10.1158/1078-0432.CCR-20-1247 32816891 PMC9210997

[160] Boudousquie C Bossi G Hurst JM: Polyfunctional response by ImmTAC (IMCgp100) redirected CD8^+^ and CD4^+^ T cells. *Immunology.* 2017; 152(3): 425–38. 10.1111/imm.12779 28640942 PMC5629433

[161] Liddy N Bossi G Adams KJ: Monoclonal TCR-redirected tumor cell killing. *Nat Med.* 2012; 18(6): 980–7. 10.1038/nm.2764 22561687

[162] Nowis D Makowski M Stokłosa T: Direct tumor damage mechanisms of photodynamic therapy. *Acta Biochim Pol.* 2005; 52(2): 339–52. 10.18388/abp.2005_3447 15990919

